# A Supine Position and Dual-Dose Applications Enhance Spray Dosing to the Posterior Nose: Paving the Way for Mucosal Immunization

**DOI:** 10.3390/pharmaceutics15020359

**Published:** 2023-01-20

**Authors:** Amr Seifelnasr, Mohamed Talaat, Pranav Ramaswamy, Xiuhua April Si, Jinxiang Xi

**Affiliations:** 1 Department of Biomedical Engineering, University of Massachusetts, Lowell, MA 01854, USA; 2 Department of Mechanical Engineering, California Baptist University, Riverside, CA 92504, USA

**Keywords:** nasal spray, liquid film translocation, supine position, wall liquid-holding capacity, nasopharynx, mucosal immunization

## Abstract

Delivering vaccines to the posterior nose has been proposed to induce mucosal immunization. However, conventional nasal devices often fail to deliver sufficient doses to the posterior nose. This study aimed to develop a new delivery protocol that can effectively deliver sprays to the caudal turbinate and nasopharynx. High-speed imaging was used to characterize the nasal spray plumes. Three-dimensional-printed transparent nasal casts were used to visualize the spray deposition within the nasal airway, as well as the subsequent liquid film formation and translocation. Influencing variables considered included the device type, delivery mode, release angle, flow rate, head position, and dose number. Apparent liquid film translocation was observed in the nasal cavity. To deliver sprays to the posterior nose, the optimal release angle was found to be 40° for unidirectional delivery and 30° for bidirectional delivery. The flow shear was the key factor that mobilized the liquid film. Both the flow shear and the head position were important in determining the translocation distance. A supine position and dual-dose application significantly improved delivery to the nasopharynx, i.e., 31% vs. 0% with an upright position and one-dose application. It is feasible to effectively deliver medications to the posterior nose by leveraging liquid film translocation for mucosal immunization.

## 1. Introduction

Respiratory infectious diseases often enter the human body through the nose. SARS-CoV-2 preferentially binds to the ACE2-rich tissue cells in the posterior nose. Coronavirus can quickly replicate in the nasal mucosa, which has made it a frequent sampling site (i.e., for nasal swabs) in COVID-19 screening. Evidence has shown that viral binding and replication start in ciliated epithelial cells after being infected with virus-laden aerosols, and this process can last for 3~14 days without obvious symptoms [1]. The binding affinity between the coronavirus’ S-protein and angiotensin-converting enzyme 2 (ACE2) is the crucial factor for viral transmission, while ACE2 is highly populated in nasal epithelial cells [2]. Lee et al. [3] mapped the distribution of subcellular ACE2 in human nasal epithelium. They observed the highest expression of ACE2 in ciliated epithelial cells and in descending order in goblet, club, and suprabasal cells. Moreover, the goblet cell in the nose is one of the three verified entry locations for SARS-CoV-2, which needs two coexisting proteins: ACE2 and type II transmembrane serine protease (TMPRSS2) [4,5]. The anterior one-third of the nose is lined with the stratified squamous epithelium, while the posterior two-thirds of the nose and nasopharynx are lined with mucus-producing goblet cells, columnar ciliated cells, and the suprabasal membrane [6].

Nasal sprays have been frequently used to administer influenza vaccines [7]. The elevated concentration of ACE2 in the posterior nose indicates that intranasal vaccines need to be dispensed to the posterior two-thirds of the nose and nasopharynx to be efficacious in eliciting mucosal immunization [8,9]. However, conventional nasal sprays fail to deliver drugs to the caudal turbinate and nasopharynx. Due to the geometrical complexity, most of the applied doses are deposited in the nasal vestibule and nasal valve, with only a small fraction passing the nasal valve filtration and reaching the turbinate region [10,11,12,13]. Many factors can contribute to the low doses in the posterior nose, for instance, spray properties, breathing conditions, the delivery method, and the nasal physiology [14]. Nasal spray properties that influence intranasal dosimetry include, but are not limited to, the spray discharge velocity, aerosol mean diameter and distribution, spray plume angle, release angle to the nostril, applied dose, and number of applications. The mean diameter of nasal spray droplets is much larger than those of nebulizers and metered dose inhalers, which range between 20 and 300 µm and whose deposition is predominantly governed by inertial impaction [15]. Cheng et al. evaluated the dose distributions from various nasal pumps in nasal casts reconstructed from magnetic resonance imaging (MRI) [16]. They found that wide plumes and/or large droplets give rise to higher deposition in the anterior nose, whereas narrow plume angles and/or small droplets enhance delivery to the turbinate region. Kundoor and Dalby studied the effects of the spray release angle in the range of 0°–90° with an increment of 15° [17]. The optimal release angle was found to be 60° in delivering the sprays to the middle turbinate. Liquid film translocation can also affect the dosimetry distribution because a liquid film can form, grow, and move from the accumulation of deposited droplets. It has been shown that liquid film translocation can substantially enhance the dosing to the olfactory cleft by adopting a head-to-floor position [18]. To achieve optimal protection against COVID-19 infection, a nasal device that can effectively deliver high doses of nasal sprays to the posterior nose is needed [19,20,21,22,23,24,25].

The objective of this study was to develop a method that can effectively deliver nasal sprays to the caudal turbinate and nasopharynx for mucosal immunization against infectious respiratory diseases. Various delivery parameters were tested in an image-based nasal airway model to understand the mechanisms underlying the liquid film translocation and identify the optimal delivery protocol. Specific aims included the following:

(1) Visualize the spatiotemporal development of the nasal sprays from different devices using a high-speed camera.
(2) Visualize the liquid film translocation in the nose with unidirectional delivery for various administration angles, head positions, and inhalation flow rates.
(3) Visualize the liquid film translocation with bidirectional delivery for various administration angles, head positions, inhalation flow rates, and the number of applications.
(4) Quantify the nasal spray deposition in the front nose, turbinate, and nasopharynx.
(5) Compare the performances among test cases and identify the optimal combination of the nasal device, delivery method, administration angle, inhalation flow rate, head position, and number of spray applications.

## 2. Materials and Methods

### 2.1. Study Design

Nasal spray delivery can be influenced by a variety of parameters (i.e., device, drug, patient, device, administration, and formulation related), and no individual parameter is universally accepted as a predictive index for the dosimetry distribution within the nasal cavity, making in vitro testing a necessity in studying nasal spray dosimetry. To evaluate the feasibility of delivering clinically significant doses to the posterior nose, the nasal dosimetry sensitivity to key influencing factors was tested in this study, including nasal devices, the delivery method, the administration angle, the inhalation flow rate, the head position, and the number of applications.

Three nasal spray devices were initially considered to select a candidate most favorable for posterior nose delivery: a saline nasal spray (Basic Care, with purified water, 0.65% sodium chloride, disodium phosphate, phenylcarbinol, monosodium phosphate, and benzalkonium chloride), the Xlear nasal spray (Xlear, with purified water, 11% xylitol, 0.85% sodium chloride, and grapefruit seed extract), and a refillable soft-mist spray inhaler (Hengni, with purified water and 0.9% sodium chloride). To this aim, smaller droplet sizes and lower existing speeds were preferred. Because spray actuation is a highly transient process, high-speed imaging techniques were used to visualize the spray release and subsequent plume development.

**Figure 1:**
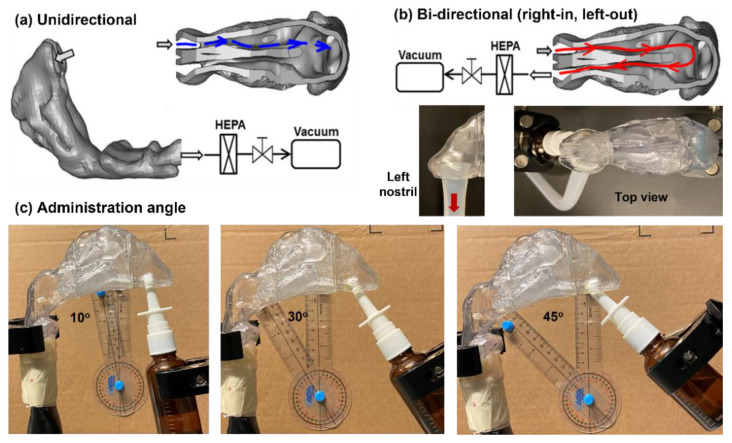
Nasal spray delivery diagram in (**a**) unidirectional mode, (**b**) bidirectional mode, and (**c**) administration angle to the right nostril: 10°, 30°, and 45°. To quantify the regional doses, the nasal cavity was divided into three parts: front nose, turbinate, and nasopharynx.

It was hypothesized that bidirectional delivery could deliver more medications to the caudal turbinate and nasopharynx [26]. As such, the dosimetry of the nasal sprays was compared between these two delivery methods for different respiration flow rates and spray release angles. A baseline respiration flow rate of 30 L/min was implemented in the conventional unidirectional delivery, while a baseline flow rate of 11 L/min was used in bidirectional delivery. This difference was because the unidirectionally inhaled flow included flows through both nasal passages, while the bidirectional flow was the same flow that entered one nostril and exited from the other. A vacuum (Robinair 3 CFM, Warren, MI, USA) was connected to the nasopharynx to generate inhalation flows for unidirectional delivery and to the left nostril for bidirectional delivery, as illustrated in Figure 1a and Figure 1b, respectively. The inhalation flow rate was controlled using an inline flow meter (Omega, FL-510, Stamford, CT, USA). Considering that the spray-releasing angle could significantly affect the dosimetry mapping, five releasing angles ranging from 10° to 45° from the nostril normal were studied (Figure 1c).

To study the head orientation effects, both the upright and supine positions were considered. To study the dosimetry after multiple applications, the nasal spray was applied either once or twice. The delivered doses were quantified by weighing the mass difference before and after spray administration. Regional deposition rates in the vestibule, turbinate, and nasopharynx were quantified and compared among test cases.

### 2.2. Nasal Airway Casts and Computational Model

A nasal airway model previously developed by Xi et al. [27] was used in this study. Briefly, the airway model was segmented from the MRI of a 53-year-old male (512 × 512-pixel resolution). A hollow-cast model was prepared using Magics (Materialise, Ann Abor, MI, USA) with a constant layer thickness of 4 mm and a feature detail size of 0.1 mm. Hollow-cast replicas were manufactured using transparent stereolithography (SLA) material (Somos WaterShed XC 11122) and a Stratasys Polyjet printer (Wenext, Shenzhen, China). A major advantage of using a transparent cast was that the instantaneous transport of the nasal sprays within the nasal cast could be visualized. To quantify the regional dosimetry, the hollow cast was divided into three components: front (nasal vestibule and valve), middle (turbinate), and back (nasopharynx). A step-shaped connector was created at the interface of each component, which had a width of 2.0 mm (half the wall thickness), a height of 2.5 mm, and a clearance of 0.2 mm so that the two parts could easily connect with each other. Transparent tape (Silicon Scar Tape) was applied to the interfaces to minimize the probability of leakage.

### 2.3. Visualization of Spray Plumes in Open Space

To understand the nasal spray patterns, a high-speed camera (Phantom VEO 1310, up to 11,000 fps acquisition rate) was used to visualize the dispersion of the spray plumes after being discharged into an open space. An LED light with up to 41,500 lumens (Mankerlight MK38) was adopted to illuminate the spray plumes. The acquisition speeds of the high-speed camera were varied in the range of 2000–6000 fps to select the best visualization effects in both the spatial and the temporal dispersion of the spray plumes [28].

### 2.4. UV-Illuminated Fluorescent Visualization of Spray Dynamics within the Nose

The behavior and fate of spray droplets in the nasal cast were visualized using 0.5% green ultraviolet (UV) reactive water-soluble fluorescent dye (GLO Effex, Murrieta, CA, USA) and a 385–395 nm LED light. Considering that liquid film translocation can be affected by surface smoothness (or roughness), a thin layer of silicone oil (MicroLubrol PMS-0125 Phenyl Methyl Silicone Oil, 125 CST Viscosity) was applied on the inner surface to simulate the mucus layer of human nasal airways. All tests were conducted in a dark room.

### 2.5. Regional Dose Quantification

The delivered doses in the front/middle/back of the nose were quantified using the weight difference (ΔW = W_1_ − W_0_), and the deposition fraction was quantified as the ratio of ΔW to the applied dose [13]. The weight of the sectional cast was measured immediately before and after drug delivery, i.e., W_0_ and W_1_. A high-precision electronic scale (120 g/0.0001 g, Bonvoisin, A&D Medical, San Jose, CA, USA) was used to measure the weight. Photos of the deposition distribution with fluorescent dyes were taken in the transparent airway model. Each test case was repeated at least three times to evaluate the dosimetry variability.

### 2.6. Statistical Analysis

Minitab (State College, PA, USA) was used to conduct associated statistical analysis. One-way analysis of variance (ANOVA) was used to evaluate sample variability. The delivered doses were expressed as the mean ± standard deviation.

## 3. Results

### 3.1. High-Speed Imaging of Soft-Mist and Squeeze-Bottle Sprays

**Figure 2:**
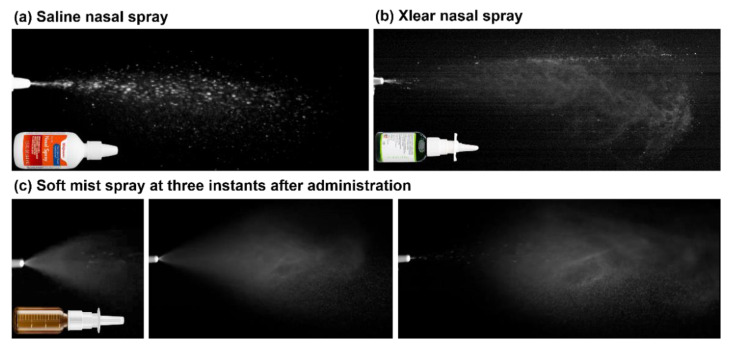
High-speed imaging of the three types of nasal sprays: (**a**) saline nasal spray, (**b**) Xlear, and (**c**) soft-mist spray (at three instants after administration).

Figure 2a,b shows the spray generation and transport from two conventional nasal devices: a saline nasal spray and Xlear, respectively. The saline nasal spray generated much larger droplets; the droplet motions appeared to be predominately moving straightforward, with no apparent vortices (Figure 2a). The droplets from the Xlear nasal spray were apparently smaller in size than those from the saline nasal spray; the spray plume gradually developed into vortex flows (Figure 2b). However, large droplets were also observed immediately downstream of the Xlear spray nozzle (Figure 2b). High-speed images of the soft-mist nasal spray are shown in Figure 2c at varying instants after actuation, which generated smaller droplets with a more homogeneous size distribution. The droplet size distribution of the soft-mist spray was measured at 3 cm from the nozzle using the Malvern Spraytec, with D10 being 24.5 ± 0.3 µm (i.e., 10% of the sample is smaller than 24.5 ± 0.3 µm), D50 being 43.9 ± 0.7 µm, and D90 being 75.8 ± 2.9 µm. Considering that large droplets and straight droplet trajectories would lead to deposition mainly in the front nose from inertial impaction, the soft-mist spray bottle was chosen in the following investigations based on the rationale that smaller aerosols could reduce the front-nose deposition and increase the spray dispensing beyond the nasal valve.

### 3.2. Unidirectional Delivery

**Figure 3:**
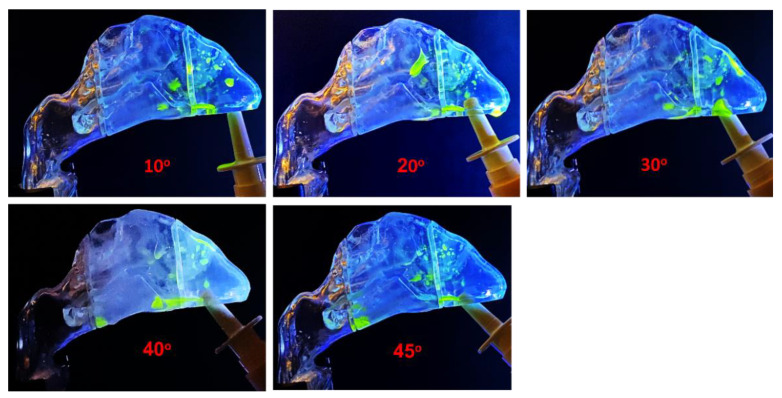
Nasal spray deposition distribution using the unidirectional delivery method for different administration angles: 10°, 20°, 30°, 40°, and 45°.

Figure 3 shows nasal spray deposition using the unidirectional delivery method with five device orientations from the vertical direction (i.e., spray release angles). For all device orientations considered, the majority of the nasal sprays deposited in the front nose, especially in the nasal valve region. Among them, the 40° orientation gave the optimal dose in the turbinate region. However, no perceivable dose was delivered to the nasopharynx.

Liquid film translocation was apparent in the nasal cavity during the nasal spray application. At 10° orientation, the nasal sprays were predominately deposited in the nasal vestibule, particularly the roof of the vestibular. There was clear liquid dripping downward due to gravity, as a significant dose of spray droplets was concentrated in this region. These droplets coalesced into patches of liquid films, and the self-weight of certain patches became large enough to overcome the surface tension and wall friction, causing the liquid film to drip along the gravitational direction. Liquid film translocation due to the flow shear stress at the air–liquid interface was also observed. In the case of 40° orientation, a thin liquid film moved from the middle turbinate toward the caudal turbinate, which eventually led to the formation of a large drop on the nose floor at the caudal turbinate (40°, Figure 3).

### 3.3. Bidirectional Delivery

#### 3.3.1. Effects of Spray Administration Angles (Relative to the Nostril)

**Figure 4:**
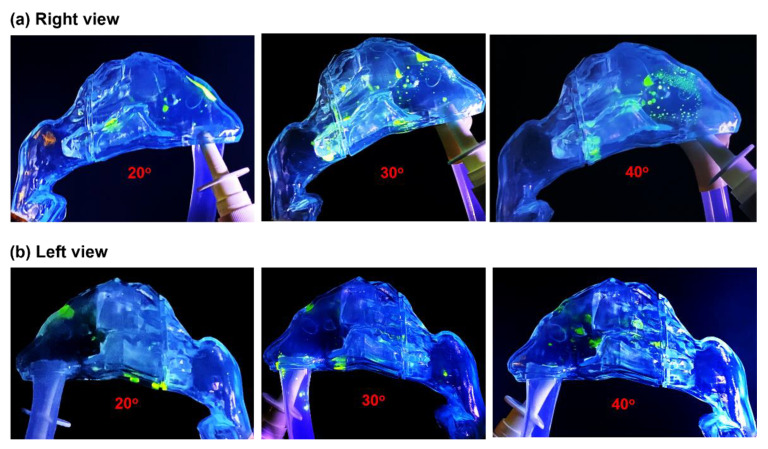
Nasal spray deposition distribution using the bidirectional delivery method for different administration angles: 20°, 30°, and 40°. (**a**) Right view and (**b**) left view. Enhanced deposition in the turbinate region was observed with an administration angle of 30°.

The nasal spray deposition using bidirectional delivery is shown in Figure 4 for different device orientation angles at a flow rate of 11 L/min. Considering that the sprays can deposit in both nasal passages using bidirectional delivery, photos were taken from both sides of the nose. The upper row in Figure 4 shows the right passage, into which the spray was applied; the lower row shows the left passage, where deposition occurred from the convection of small droplets and liquid translocation. Overall, the bidirectional delivery method delivered more doses beyond the nasal valve and to the middle and caudal turbinate. At a device orientation of 30°, a certain dose was also observed in the nasopharynx, which was not observed using unidirectional delivery. Moreover, different deposition patterns were observed between the bidirectional and unidirectional delivery methods (Figure 4 vs. Figure 3). This could be attributed to different flow dynamics, vortex fields, and associated wall shear (liquid-film-stabilizing force) and flow shear (destabilizing force), with more detailed explanations presented in Section 4.2.

Regarding the left passage, only a small quantity of nasal spray was observed in the posterior nose. This was because of liquid translocation either along the septum (middle wall) or channeled by the middle or inferior turbinate. Both turbinate projections resemble V-shaped open conduits, and the liquid within them is driven by the liquid self-weight or flow shear. Note that UV fluorescence was only visible when the UV concentration was high enough.

**Figure 5:**
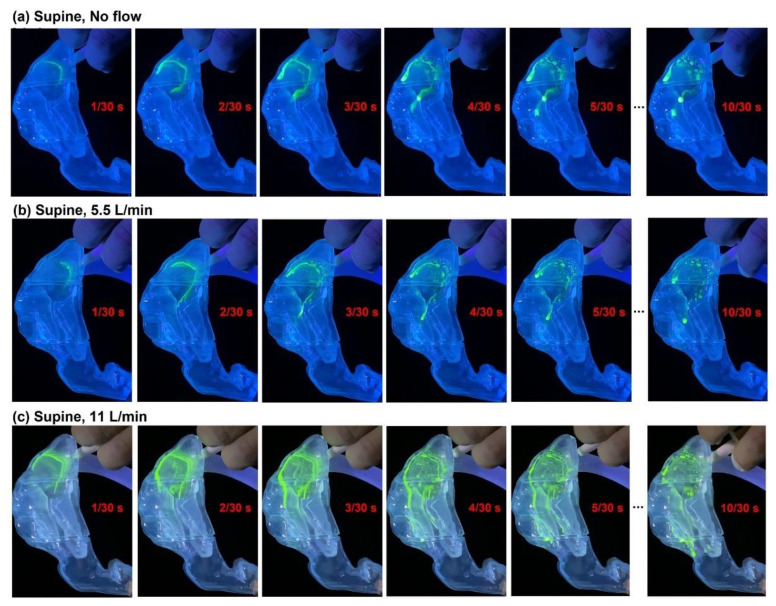
Liquid film translocation vs. time after administration using bidirectional delivery mode in a supine position under varying respiration flow rates: (**a**) no flow, (**b**) 5.5 L/min, and (**c**) 11 L/min.

To evaluate the flow shear at the air–liquid interface, the dynamics of the liquid film during the first 0.33 s were compared in Figure 5 among three respiration flow rates (i.e., 0, 5.5, and 11 L/min). Clearly, a flow rate of 11 L/min and the resultant shear force exerted a more significant effect on the wall film stability and translocation compared to 5.5 L/min and 0 L/min. At 11 L/min, the nasal spray spread a longer distance at any instant considered herein than at 5.5 L/min and 0 L/min. However, more similarities were observed between 0 L/min and 5.5 L/min. Both showed a slow progression in spray deposition from a C-shaped strip to a horseshoe to a closed loop, which eventually merged into a dripping drop (Figure 5a,b). The drop stabilized in the middle turbinate, as illustrated by the negligible change in the deposition pattern from 5/30 s to 10/30 s. These similarities indicated that the flow shear at 5.5 L/min and gravity were not large enough to mobilize the liquid film to move much, despite a slightly longer distance of the film spreading than without a flow shear (last column, Figure 5b vs. Figure 5a). Conversely, the significantly enhanced liquid film spreading at 11 L/min indicated that the flow shear at 11 L/min succeeded in destabilizing the liquid film, which together with gravity surpassed the stabilizing forces from viscosity and surface tension. It was also observed that the mobilized wall film at 11 L/min stopped after traveling a certain distance and reached a new stabilized condition due to the decreasing liquid film thickness and gravity as it spread to other regions.

#### 3.3.2. Effects of Dual-Spray Applications on Dosimetry

**Figure 6:**
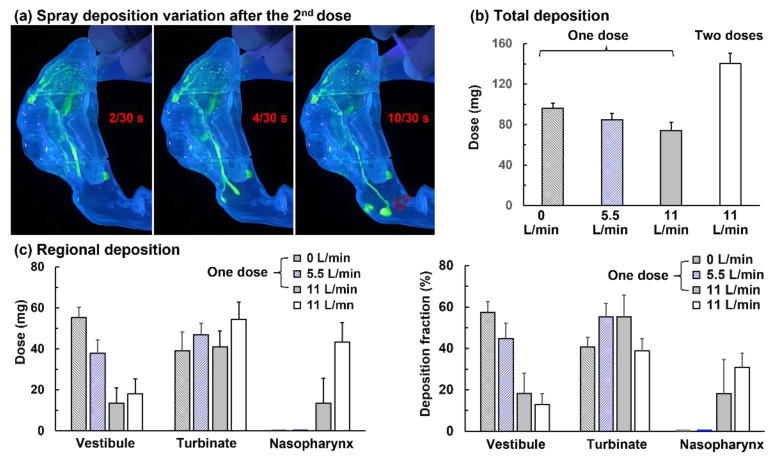
Nasal spray deposition with single- and dual-spray applications administered bidirectionally in a supine position: (**a**) liquid film translocation after applying two doses at a flow rate of 11 L/min, (**b**) total deposition, and (**c**) regional deposition at different flow rates.

The airway wall has a limit to the amount of liquid it can hold, and this liquid-holding capacity varies with the slope and roughness of the wall. It is conjectured that a second application will deliver a higher percentage of the applied dose to the target. Because the first application has fulfilled the liquid-holding capacity of the airway wall leading to the target, all (or most of) the second dose should follow the same path and translocate to the target, with no (or negligible) loss to these walls. Figure 6a shows the liquid film translocation after the second dose when administered bidirectionally in a supine position and at a flow rate of 11 L/min. Apparently, more doses were found in the nasopharynx. As the first dose paved the way in the front nose and middle turbinate, the extra liquid mass streamed downward following the furrow between the inferior and the middle turbinate (referred to as turbinate furrow thenceforth; Figure 6a). This led to a large drop of sprays in the nasopharynx, which stabilized on the flat nasopharynx wall (0.33 s, Figure 6a).

A comparison of spray dosimetry among different flow rates, as well as between one and two doses, is shown in Figure 6b,c for total and regional deposition, respectively. The total deposition gradually decreased as the flow rate increased from 0 to 5.5 to 11 L/min (Figure 6b). This was as expected, considering that stronger convection would carry away more aerosol droplets out of the airway. Considering the regional deposition, the deposited mass in the anterior nose (vestibule) drastically decreased from 0 to 5.5 to 11 L/min, reflecting more intensified liquid film translocation with higher flow shear (Figure 6c). Insignificant variations in the turbinate deposition were observed among the three flow rates, as the extra mass beyond the vestibular liquid-holding capacity moved to the turbinate region, driven by gravity (and flow shear for 5.5 L/min and 11 L/min). No deposited mass was found in the nasopharynx when the flow shear was absent (0 L/min) or small (5.5 L/min), while a dose of 13.5 mg (or 18.2% of the total deposition) was found when the flow rate increased to 11 L/min (Figure 6c).

A second dose approximately doubled the total deposition at 11 L/min (Figure 6b) but more than tripled the deposition in the nasopharynx (left panel, Figure 6c). The fact that the first dose paved the way to the target and fulfilled the wall liquid-holding capacity made it easier for the second dose to reach the target with more spray mass than was otherwise needed to saturate the liquid film. This was evidenced by the deposited masses in the vestibule and turbinate (left panel, Figure 6c), which was only slightly higher in dual-dose vs. one-dose application for a given flow rate (11 L/min). As a result, a higher percentage of the total dose was delivered to the nasopharynx with dual-dose than with one-dose administration (30.9% vs. 18.2%; right panel Figure 6c; *p*-value = 0.044).

#### 3.3.3. Head Positions (Supine)

**Figure 7:**
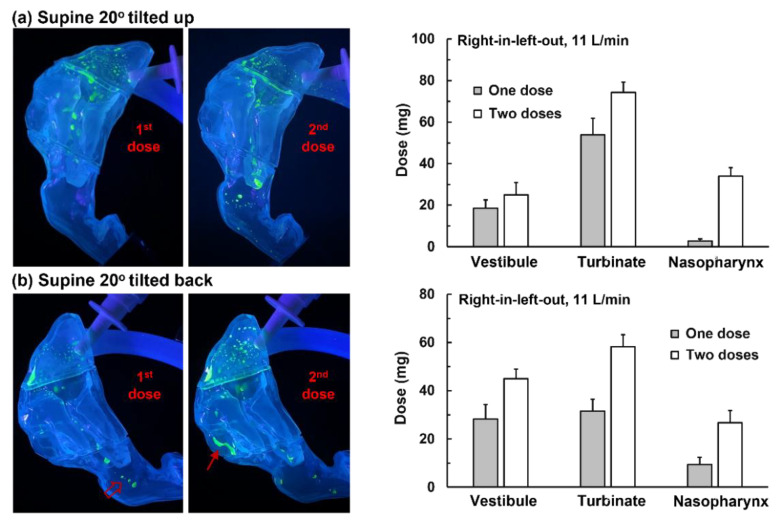
Head orientation effects on nasal spray deposition distribution: (**a**) supine 20° tilted up (head on a pillow) and (**b**) supine 20° tilted back.

Considering that the liquid-holding capacity of the turbinate furrows is sensitive to the head orientation, two head positions were evaluated that tilted up and down from the flat supine position by 20°, respectively. As shown in Figure 7a, when tilting the head up by 20° (equivalent to the head on a pillow), most of the spray droplets were deposited in the inferior turbinate and nasal floor. This was because the vestibule was aligned with the inferior turbinate along gravity in this case. Moreover, the liquid film spread a shorter distance than in the supine position because tilting the head up by 20° changed the turbinate furrow from vertical to a 70° slope. Applying the second dose delivered significantly more doses to the caudal turbinate, but a negligible dose was observed in the nasopharynx, as displayed in the middle panel of Figure 7a. The posterior nose dose (i.e., caudal turbinate and nasopharynx) with one-dose and dual-dose applications was 2.9 mg and 34.0 mg, respectively (right panel, Figure 7a).

Figure 7b shows the deposition distribution when the head was tilted back by 20°. Note that this would also change the turbinate furrow from vertical to a 70° slope, but the liquid film would touch the other side of the turbinate furrow and have a different liquid film stability. With one applied dose, the liquid film traveled down the turbinate furrow, with several drops reaching the nasopharynx (hollow red arrow, left panel, Figure 7b).

Applying another dose delivered more sprays to the caudal turbinate, particularly to the ridge of the caudal turbinate (solid red arrow, middle panel, Figure 7b). The turbinate deposition increased from 31.5 mg with one-dose application to 58.3 mg with dual-dose application; the nasopharynx deposition increased from 9.4 mg with one-dose application to 26.7 mg with dual-dose application (right panel, Figure 7b). Based on these observations, it was inferred that three factors are necessary for enhanced delivery to the posterior nose: (1) flow shear mobilizing the liquid film, (2) gravity aligning with the target, and (3) highly slanted turbinate furrows assisting the liquid film motion and minimizing the wall liquid-holding capacity. As a result, adopting a supine position or tilting the head backward by 20° is supposed to effectively deliver sprays to the posterior nose (Figure 6 and Figure 7b).

### 3.4. Revisiting Unidirectional Delivery

#### Effects of Spray Administration Angles (Relative to the Nostril)

It was noted that these aforementioned three factors needed for enhanced delivery to the posterior nose could be valid for both bidirectional and unidirectional delivery methods as long as the flow shear is sufficient in mobilizing the liquid film. Thus, unidirectional delivery in a supine position with a high flow rate and multiple-dose applications could also give similarly enhanced doses to the target as bidirectional delivery.

**Figure 8:**
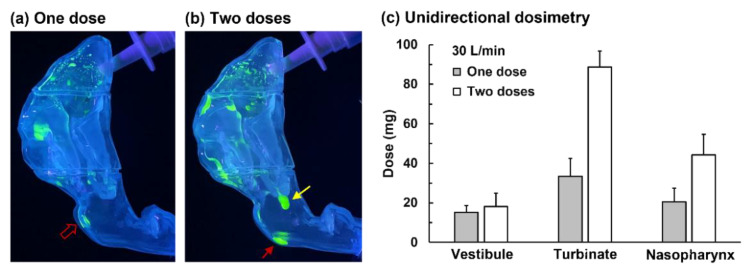
Nasal spray deposition distribution using unidirectional delivery in a supine position with a flow rate of 30 L/min with (**a**) one dose, (**b**) two doses, and (**c**) quantification of regional deposition fractions.

Figure 8a,b displays the deposition pattern of unidirectional delivery at 30 L/min with one dose and two doses, respectively. The supine position appeared to significantly offset the liquid-film-stabilizing forces, which made it easier for the flow shear to mobilize the wall film. The increased gravitational component and reduced wall liquid-holding capacity in the supine position also facilitated the liquid film translocation toward the back of the nose, as illustrated by the increased nasopharynx dose (hollow red arrow, Figure 8a).

Applying a second dose further enhanced deposition in the nasopharynx (red arrow, Figure 8b). Moreover, liquid dripping was observed at the caudal tip of the turbinate (yellow arrow, Figure 8b), indicating that the liquid washed through the turbinate region. Quantitatively, applying a second dose more than doubled the deposition in the turbinate region (i.e., from 39.0 to 88.8 mg, *p*-value = 0.016) and nasopharynx (i.e., from 8.6 to 32.6 mg, *p*-value = 0.031), as shown in Figure 8c. It was also noted that the same factors (gravity and flow shear) that helped mobilize the liquid film could also reduce the maximal liquid film thickness, thus slightly decreasing the dose adhering to the same region.

## 4. Discussion

### 4.1. Has the New Delivery System Delivered Sufficient Doses to the Posterior Nose?

In this study, we sought to develop an effective protocol for delivering nasal sprays to the posterior nose by testing different delivery modes, administration angles, inhalation flow rates, and the number of applications in a progressive manner. That is, after each parametric study, not only were the performances compared but also the underlying mechanisms were examined, hypotheses were checked, and new hypotheses were planned to be tested (more details in the following Section 4.2). With this progressive examination, it was found that a supine position and dual applications are able to significantly improve the doses delivered to the caudal turbinate and nasopharynx, which are the targets for mucosal immunization. Compared to the 0~5% of deposition fractions to these two regions using conventional nasal inhalers, the new protocol managed to deliver 31% of the dose to the nasopharynx alone (Figure 6c). This significant enhancement in posterior nose delivery can pave the way for mucosal immunization, for which low doses to the target using conventional methods have been a bottleneck to their wide clinical application, even though many nasally inhaled vaccines have been actively developed during the pandemic in the past 2 years [29,30,31,32,33].

Formulation viscosity can significantly affect spray generation and the subsequent dosimetry in the nose. Previous studies have demonstrated that a more viscous formulation would generate a spray with a narrower plume angle and large droplets, leading to a smaller spray area [34]. As a result, more doses would be deposited in the vestibule and anterior turbinate in comparison to formulations with lower viscosities [35,36]. Viscosity modifiers, such as mucoadhesive polymers, have often been added to nasal spray formulations to improve local delivery or cellular uptake [37,38]. In this study, saline solution was used that had a lower viscosity, which was expected to yield more distal deposition in the nose than using actual nasal formulations. In particular, slightly lower deposition in the nasopharynx was expected in clinical practice. Considering lipophilic drugs, suspension or emulsion formulations would form, which could affect the rate of both dissolution and film translocation [39]. Han et al. [40] measured the surface tension of various oral and nasal suspension formulations and reported a lower surface tension magnitude compared to saline water. Therefore, the liquid film on the nasal wall was expected to be less stable and more prone to translocate to the back of the nose.

### 4.2. Mechanisms Underlying Successful Posterior Nose Delivery

Controlled liquid film translocation was found to be the key to the success or failure of effective delivery to the caudal turbinate and nasopharynx. For a given delivery condition (device, administration angle, head position, etc.), liquid translocation from initial deposition sites to the target would be determined by three factors: First, whether a destabilizing force could mobilize or start the translocation of the liquid film; second, the translocation direction and distance followed gravity and were channeled by the nose topology; and lastly, the translocated mass depended on the applied mass over the liquid-holding capacity of the nasal wall.

Due to the large droplet sizes and high discharging velocities, nasal sprays often deposit in the anterior nose, particularly in the vestibule and nasal valve. Because of the highly localized deposition, liquid films form from these droplets as distinct or connected patches. However, whether these liquid film patches stay or move depends on the rivalry between the stabilizing and destabilizing forces. In this regard, surface tension (both liquid–air and liquid–wall) and viscosity (static wall fraction) were stabilizing forces, while gravity (i.e., tangential components of the liquid self-weight) and flow shear (at the air–liquid interface) were destabilizing forces. In this study, we observed negligible translocation when there was no inhalation flow (thus no flow shear), with most of the nasal spray being deposited in the anterior nose and staying there (Figure 5a). Increasing the flow rate increased the film translocation in both extent and traveling distance regardless of the delivery mode (Figure 5b,c). This indicated that flow-induced shear at the air–liquid interface could be the determining factor in mobilizing the liquid film, especially in regions where the stabilizing forces from surface tension and viscosity are only slightly larger than the destabilizing gravitational force [41,42,43].

How far the liquid film could move depended on the flow shear, gravity, and nose topology along the way. The liquid-holding capacity was influenced by both the wall slope and the local wall topological details. Previous studies have reported that the maximal liquid film thickness is reversely correlated with the wall slope angle [44,45]. A rough wall with geometrical irregularities could hold more liquid due to a large surface area for the intermolecular force, as well as a physical interlocking effect, leading to a higher static wall fraction [46]. Once the liquid film is mobilized, the kinematic wall shear would be much smaller due to the lubrication effects by transiting from static interlocking to laminar layer sliding (ref-lubrication) [47]. The motion of the liquid film will accelerate when moving down a slope and slow down when working against gravity. The liquid film may also stop as it fulfills the new walls’ liquid-holding capacity while spreading until the liquid film is too thin to overcome the stabilizing forces. As a result, aligning gravity toward the target is a promising approach to maximize the delivered doses via film translocation, as demonstrated in Figure 5, Figure 6 and Figure 7. Aligning the target with gravity has often been practiced in the treatment of rhinosinusitis [48]. Merkus et al. [49] recommended a head-to-floor position to enhance nasal spray delivery to the olfactory cleft after comparing four head positions: upright, supine-tilting-back, lying-on-one-side, and head-to-floor positions. Cannady et al. [50] reported that the head-to-floor position could deliver more drugs to the ethmoid cavity, maxillary sinus, and sphenoid sinus. Similarly, Mori et al. [51] observed that the lying-on-one-side (Kaiteki) position allows more nasal drops to reach the olfactory region. However, it is also cautioned that the practice of aligning gravity to the target is not guaranteed to deliver the maximal doses to the target, because the liquid motion is also subject to the wall geometrical details that can be either a blockage or a conduit to the liquid flow.

Once a path of liquid film translocation to the target was established (by adopting a specific device, delivery mode, administration angle, head position, etc.), the dose to the target could be controlled by applying multiple doses. As shown in Figure 6, Figure 7 and Figure 8, a significantly higher dose from the second application was delivered to the posterior nose than from the first application, in the light of the fact that part of the first dose had been allocated to fill the wall liquid-holding capacity. However, too many applications would overflow the targeted regions and cause unwanted deposition in other regions [52,53].

### 4.3. Bidirectional vs. Unidirectional Modes

The experimental results in this study show that using the bidirectional route delivers higher doses to the caudal turbinate and nasopharynx than using the unidirectional route. With a single spray application, the optimal nasopharynx dose was 13.5 mg vs. 8.6 mg using the bidirectional and unidirectional methods, respectively (Figure 6c vs. Figure 8c). With a dual-dose application, the nasopharynx dose was 32.6 mg bidirectional ly vs. 43.3 mg unidirectionally (Figure 6c vs. Figure 8c).

Using a transparent nasal replica cast, it was clearly demonstrated that the liquid film translocation was far more intensified during bidirectional delivery than during unidirectional delivery, all other parameters being the same. Even though the exact reason underlying this difference was not clear at this moment, we speculated that this could be attributed to the distinct flow dynamics between these delivery methods, which further led to different wall shear (stabilizing force) and flow shear (destabilizing force). In the bidirectional route, the nasopharynx was closed and the airflow followed a U-shaped trajectory from the right nostril to the left nostril. Note that it is a natural reflex in humans that the soft palate is lifted up upon exhalation and blocks the oropharynx that connects the oral and nasal airways. As a result, the nasopharynx serves as a dead end, which generates an elevated pressure that diverts the airflow by 180° from one nasal passage to the other. It is likely that this forceful diversion in the flow direction creates a pressure-flow-shear field that is more prone to mobilizing the liquid film and driving the liquid film motion. Further studies are needed to prove/disprove this hypothesis and to unravel the exact reason responsible for the improved bidirectional dosimetry.

### 4.4. Limitations

This study can be further improved if more physiological factors can be considered, including a large cohort of nose models, more delivery scenarios, and compliant walls. Intranasal spray dosimetry is sensitive to nasal anatomical details; the inter-subject variability can be large and warrants future investigations [54,55,56,57]. It will be desirable to know the range or confidence level of the nasal dosage for a given device, formulation, and targeted patient population (e.g., child, adult, senior) [36,58,59,60]. Similarly, a limited number of delivery conditions were considered. By considering more test cases that cover the entire design space, a response surface can be developed, which can immediately provide an empirical estimation for any delivery scenarios [61]. Considering that nasal sprays generally have large droplet sizes and high exiting speeds, the inertial impaction will be the predominant deposition mechanism and the effects from compliant walls should be secondary [62,63]. Zeta potential analysis, which characterizes the droplet surface charge and is important in determining the stability of colloidal suspensions or emulsions, was not considered in this study based on the rationale that 0.9% saline water is a stable solution [64]. The droplet size distribution and physicochemical properties of the three nasal formulations were not fully characterized except the size distribution of the soft-mist nasal inhaler.

## 5. Conclusions

In this study, the feasibility of delivering high doses of nasal sprays to the caudal turbinate and nasopharynx was evaluated by experimentally testing different delivery methods, releasing angles, flow rates, head positions, and dosing numbers. The mechanisms underlying liquid film translocation were explored. Specific findings are as follows:

(1) For nasal spray delivery, liquid film translocation can be a more important factor than the initial deposition in determining the dosimetry distribution.
(2) Liquid film translocation is sensitive to the inhalation flow rate and head position.
(3) Liquid film translocation is more sensitive to the inhalation flow rate in bidirectional delivery than in unidirectional delivery and in a supine position than in the upright position.
(4) Factors favorable for posterior nose delivery include (1) flow shear mobilizing the liquid film, (2) gravity aligning with the target, and (3) slanted turbinate furrows assisting film motion and minimizing the wall liquid-holding capacity.
(5) A supine position and dual-dose application significantly enhance nasal spray deposition in the caudal turbinate and nasopharynx. A nasopharynx deposition fraction of 31% was achieved vs. no nasopharynx deposition in an upright position with a one-dose application.

## Data Availability

The data and images are available from the corresponding author (J.X.) upon request.
